# Improved algorithm for determining cable saddle pre-offsets considering the coupling effect of tower and splay saddles

**DOI:** 10.1038/s41598-022-26676-z

**Published:** 2022-12-26

**Authors:** Xiaokang Deng, Huiru Zhao

**Affiliations:** 1 School of Automobile and Traffic Engineering, Wuhan University of Science and Technology, Wuhan, 430081 China; 2 Master of Engineering Student, School of Automobile and Traffic Engineering, Wuhan University of Science and Technology, Wuhan, 430081 China

**Keywords:** Engineering, Mathematics and computing

## Abstract

By analysing the mechanical and geometrical relations between the main cable, tower, and splay saddles, and considering the coupling effect of the tower and splay saddles, an improved algorithm is proposed to determine the cable saddles pre-offsets of suspension bridges. The equilibrium relationship of the cable saddles, the compatible deformation condition, and the basic equation of the main cable shape are considered to establish several coupled non-linear equations up to 19, and the tower and splay saddle pre-offsets are obtained by solving the above equations with the Newton–Raphson method. This paper presents the initial value selection principle and the constraint conditions for solving the cable saddle pre-offsets of the plane cable suspension bridge and the calculation process ensures convergence. The calculation example demonstrates that the improved algorithm without an exact initial value can achieve excellent convergence.

## Introduction

In the suspension bridge, cable saddles are the main structures utilized to provide the main cable with support and to smoothly change the configuration of the main cable. The tower saddles, installed on top of the towers, transmit the perpendicular cable tensile force to the towers, while the splay saddles are located on top of the main cable trestles, which support, turn, and disperse the strands of the main cable^1,2^. Cable saddles are used to turn the main cables and can therefore directly constrain the deformation of the main cables^3^. If the saddles are installed in the positions for the completed bridge, there will inevitably be large unbalanced forces acting on the saddles in the unloaded state. These unbalanced forces may cause displacement of the towers and slippage of the cable strands in the saddles. Moreover, this will affect the safety of the entire bridge^4–7^. Therefore, to guarantee the safety of the suspension bridge construction, it is essential to consider the tower and splay saddles pre-offsets^8,9^.

Cable saddle pre-offset calculations for suspension bridges mostly use finite element methods or numerical analytical methods^10–13^. Compared to finite element methods, the numerical methods have a higher detail processing capability, a more explicit computational process, and a higher iterative convergence speed; thus, they are more widely applied to the calculation of suspension bridge cable saddle pre-offsets^14^.

This paper mainly researches numerical analytical methods for determining the pre-offsets of the tower and splay saddles. Ref.^15^ first calculated the cable configuration on the two sides of each tower saddle and the unbalanced tension forces on a cable segment under the condition of assuming an iterative initial value for the pre-offsets of each saddle. It then identified the relationship between the unbalanced forces and the slip stiffness of the cable saddle to perform the adjustment value of the pre-offsets and balanced the internal tension of the cable through multiple loop iterations. In Ref.^16^, a new concept—unbalanced force coefficient—was proposed to compute pre-offsets. Under the same premise as that of Refs.^15,16^ first calculated the unbalanced force coefficient and iteratively adjusted it by adding a different increment to the pre-offsets to meet the accuracy requirements. The above two methods were similar in that in both methods, the initial value of the pre-offsets was given and was continuously adjusted by iteration so that the forces of the cable saddles met the requirements to obtain the pre-offsets, and the difference was the interative initial value adjustment method. When considering the coupling effect of the tower and splay saddles, the two methods need to continuously try to calculate the balance conditions of the tower and splay saddles, which led to complex operations and low iteration efficiency. In Ref.^17^, the first step was to calculate the unstrained cable length based on the design state of the main cables under the final dead load. Then, the number of coupled nonlinear equations of the tower saddle and main cable, which reached up to 11, was erected based on the analysis of the cable saddle stress, and the pre-offsets were obtained by solving the system of equations. In Ref.^18^, the cable configuration in the unloaded state was calculated based on the principle that in the basic equation of the main cable alignment, the unstrained length of any cable segment remained constant during the structural construction and after its completion. The tower saddle’s pre-offsets were then solved. Although Refs.^17,18^ derived the nonlinear equations of the main cable saddle offsets, these algorithms aimed to solve the tower saddle pre-offsets without considering the joint action between the splay saddle and tower saddle offsets.

Based on the above research and analysis, this paper designed an improved algorithm to determine cable saddle pre-offsets. The proposed algorithm simultaneously considers the joint action of the splay and tower saddles in the calculation process. By analysing the tension on the saddle and main cable, the simultaneous equations are erected by applying the mechanical equilibrium and geometrical deformation conditions on both sides of the cable saddle^19,20^. Then the Newton–Raphson method is applied to solve the nonlinear equations and obtain the above pre-offsets. The proposed algorithm has the characteristics of a simple calculation process and efficient convergence.

## Analysis of the main cable tension

In the preliminary research process^21^, the researchers have put forward the unified catenary equation of main cable alignment of the suspension bridge and the calculation formula of the unstressed cable length with the gradients as the basic parameter.

**Figure 1:**
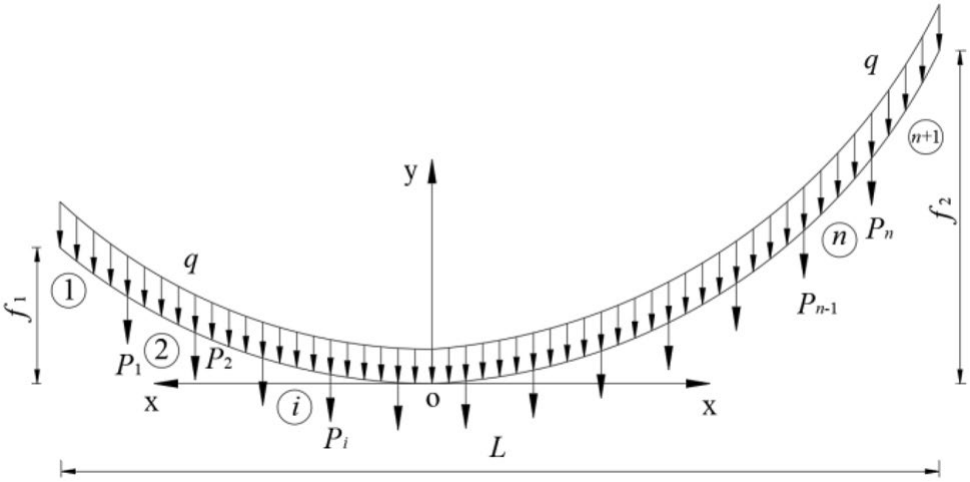
Schematic diagram of the main cable shape calculation model and cable segment division in coordinate system I.

As shown in Fig. 1, the I-coordinate system is established, with the origin at the lowest point, *O*, and the left and right half x-axes directing horizontally to the left and right, respectively, and the y-axis pointing perpendicularly upwards. Define *A*_0_ = the area of the cross section of the unstrained cable section; *q*_0_ = the distributed load of the unstrained cable section; *A* = the area of the cross section of the anamorphic cable section; *q* = the distributed load of the deformed cable section; *E* = the elastic modulus of the main cable material; *H* = the horizontal component of the tension on the main cable.

When considering the influence of the elastic extension of the main cable on its dead weight concentration, the linear equations of the main cable are unified in Eqs. (1) and (2).

1 $$x = \frac{{H^{2} z}}{{EA_{0} q_{0} }} + \frac{H}{{q_{0} }}\ln \left( {z + \sqrt {1 + z^{2} } } \right)$$

2 $$y = \frac{{H^{2} z^{2} }}{{2EA_{0} q_{0} }} + \frac{H}{{q_{0} }}\sqrt {1 + z^{2} } - \frac{H}{{q_{0} }}$$

Then, the unstrained length of any cable segment *i* can be calculated by the undermentioned equation:

3 $$s_{0} \left( i \right) = \frac{H}{q}\left[ {z_{H} \left( i \right) - z_{L} \left( i \right)} \right]$$

where *z*_*H*_(*i*) and *z*_*L*_(*i*) are the gradients at the highest and lowest points of any cable segment *i*, respectively.

## Selection of balance conditions for tower and splay saddles

The cable saddle pre-offsets in the unloaded state enable both sides of the saddles to have a certain equilibrium relation.

A detailed analysis of the ideal equilibrium conditions of the cable saddle was performed in Ref.^22^ and it was considered that the equilibrium conditions of the tower saddles should fulfil the undermentioned requisites: (1) the tension of the main cable on the two sides of each tower saddle are equal; (2) the horizontal components of the main cable force on the two sides of each tower saddle are equal; (3) the projection of the main cable tension along the sliding surface of the saddle are equal. For common tower saddles, it can be guaranteed that the projection of the main cable tension along the sliding surface of the saddle is equal because of the horizontality of the tower. Therefore, this paper selects the second requirement to construct the following equations.

For domestic suspension bridges, there are two types of splay saddles: pendulum-type and roller-type saddles, and their structures are similar. Due to space limitations, the roller-type splay saddles (sliding splay saddles)^23^ are used in the calculation and analysis of this paper. Referring to the method described in Ref.^24^, the equilibrium condition of equal component forces of the main cables on both sides of the cable saddle along the sliding plane is selected for the analysis. The calculation method of the pendulum-type splay saddle is similar to the roller-type saddle and the difference is that the bending moment balance^25^ is used in the former equilibrium state.

## Establishment of pre-offset equations considering the coupling effects of the tower and splay saddles

**Figure 2:**
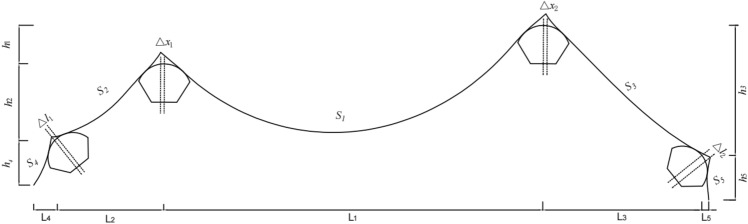
Schematic diagram of the overall calculation of the cable saddle pre-offsets.

Taking single circular curved cable saddles as an example, the schematic of the determination of the pre-offsets of the tower and splay saddles is shown in Fig. 2. The following parameters are all known conditions in the completed state of the bridge.

Define: *L*_1_ = the horizon distance between the fixed points of the main cable saddle on the left and right sides of the main span; *h*_1_*, S*_1_ = the perpendicular distance and the unstrained length of the above section respectively; *γ*_*i*_ (*i* = 1*,*2) = the angle between the line connecting the fixed point of the main cable saddle with the centre of the circle and the perpendicular line passing through the centre of the circle; *R*_1_ = the radius of the main cable saddle.

Define: *L*_*i*_(*i* = 2*,*3) = the horizon distance from the fixed point of the main cable saddle of the side span to the fixed point of the splay saddle; *h*_*i*_(*i* = 2*,*3) , *S*_*i*_(*i* = 2*,*3) = the perpendicular distance and the unstrained length of the above section respectively; *γ*_*i*_(*i* = 3*,*4) = the angle between the line connecting the fixed point of the splay saddle with the centre of the circle and the perpendicular line passing through the centre of the circle; *R*_2_ = the radius of the splay saddle.

Define: *L*_*i*_(*i* = 4,5) = the horizon distance from the fixed point of the splay saddle of the anchor span to the anchor point; *h*_*i*_(*i* = 4*,*5) , *S*_*i*_(*i* = 4*,*5) = the perpendicular distance and the unstrained length of the above section respectively; *φ*_*i*_(*i* = 1*,*2) = the angle of the sliding surface of the splay saddle.

**Figure 3:**
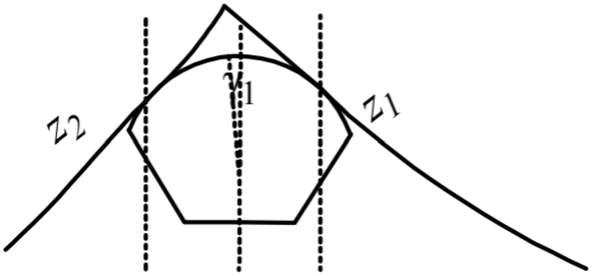
Schematic diagram of the parameters on the left tower saddle.

**Figure 4:**
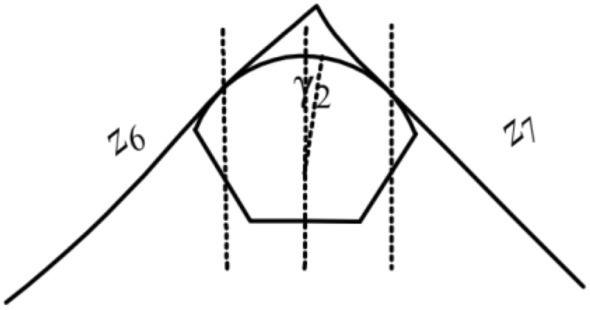
Schematic diagram of the parameters on the right tower saddle.

**Figure 5:**
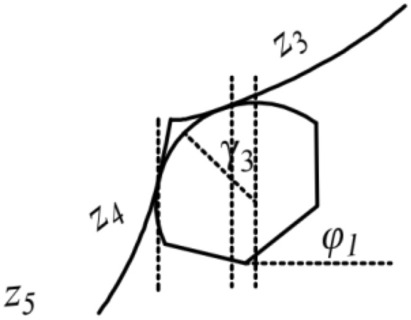
Schematic diagram of the parameters on the left splay saddle.

**Figure 6:**
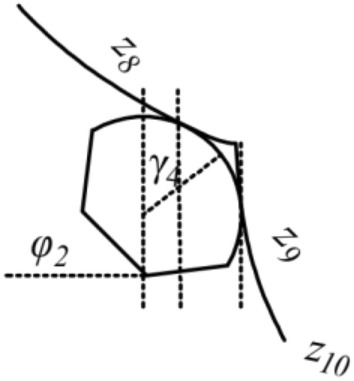
Schematic diagram of the parameters on the right splay saddle.

There are nineteen unknown parameters of the full bridge. Define: *z*_1_ and *z*_2_ as the tangent slope of the left main saddle on the main span and side span sides, separately; *z*_3_ and *z*_4_ as the tangent slope of the left splay saddle on the side span and anchor span sides, separately; *z*_5_ as the tangent slope of the main cable at the left anchor point; *z*_6_ and *z*_7_ as the tangent slope of the right main saddle on the main span and side span sides, separately; *z*_8_ and *z*_9_ as the tangent slope of the right splay saddle on the side span and anchor span sides, separately; *z*_10_ as the tangent slope of the main cable at the right anchor point; *H*_1_, *H*_2_, *H*_3_, *H*_4_,and *H*_5_ as the horizon tension of the main cable on the main span, side span, and anchor span sides, separately (especially in the unloaded state); Δ*x*_*i*_(*i* = 1*,*2) and Δ*l*_*i*_(*i* = 1*,*2) as the pre-offsets of the main saddle and the splay saddle along the sliding surface in the unloaded state compared to the completed state, respectively. The schematics of the above parameters are shown in Figs. 2, 3, 4,5,6.

Expressing the angles as radians, for the main span:

4 $$\begin{gathered} \frac{{H_{1}^{2} z_{1} }}{EAq} + \frac{{H_{1} }}{q}\ln \left( {z_{1} + \sqrt {1 + z_{1}^{2} } } \right) + R_{1} \left( {\sin \gamma_{1} + \sin \arctan z_{1} } \right){ + } \hfill \\ \frac{{H_{1}^{2} z_{6} }}{EAq} + \frac{{H_{1} }}{q}\ln \left( {z_{6} + \sqrt {1 + z_{6}^{2} } } \right) + R_{1} \left( {\sin \gamma_{2} + \sin \arctan z_{6} } \right) = L_{1} + \Delta x_{1} { + }\Delta x_{2} \hfill \\ \end{gathered}$$

5 $$\frac{{H_{1} z_{1} }}{q} + R_{1} \left( {\gamma_{1} + \arctan z_{1} } \right){ + }\frac{{H_{1} z_{6} }}{q} + R_{1} \left( {\gamma_{2} + \arctan z_{6} } \right) = S_{1}$$

6 $$\begin{gathered} \frac{{H_{1}^{2} z_{6}^{2} }}{{2EAq}} + \frac{{H_{1} }}{q}\sqrt {1 + z_{6}^{2} } - \frac{{H_{1}^{2} z_{1}^{2} }}{{2EAq}} - \frac{{H_{1} }}{q}\sqrt {1 + z_{1}^{2} } + \hfill \\ R_{1} \left( {\cos \gamma _{2} - \cos \arctan z_{6} } \right) - R_{1} \left( {\cos \gamma _{1} - \cos \arctan z_{1} } \right) \hfill \\ = h_{1} \hfill \\ \end{gathered}$$

Where $\frac{{H_{1}^{2} z_{1} }}{EAq} + \frac{{H_{1} }}{q}\ln \left( {z_{1} + \sqrt {1 + z_{1}^{2} } } \right)$,$\frac{{H_{1}^{2} z_{1}^{2} }}{2EAq}{ + }\frac{{H_{1} }}{q}\sqrt {1 + z_{1}^{2} } - \frac{{H_{1} }}{q}$ and $\frac{{H_{1} z_{1} }}{q}$ represent the horizontal distance ,the perpendicular distance and the unstrained length from the tangent point of the main cable saddle to the lowest point of the main span, calculated by Eqs. (1), (2) and (3), respectively.(The method of determining the lowest position of catenary when the main tower is of unequal height has been given in Ref.^21^.)

The same can be obtained for the left side span:

7 $$\begin{gathered} \frac{{H_{2}^{2} z_{2}^{2} }}{{2EAq}} + \frac{{H_{2} }}{q}\sqrt {1 + z_{2}^{2} } - \frac{{H_{2}^{2} z_{3}^{2} }}{{2EAq}} - \frac{{H_{2} }}{q}\sqrt {1 + z_{3}^{2} } + \hfill \\ R_{1} \left( {\cos \gamma _{1} - \cos \arctan z_{2} } \right) + R_{2} \left( {\cos \arctan z_{3} - \cos \gamma _{3} } \right) \hfill \\ = h_{2} + \Delta l_{1} \sin \varphi _{1} \hfill \\ \end{gathered}$$

8 $$\begin{gathered} \frac{{H_{2}^{2} z_{2} }}{{EAq}} + \frac{{H_{2} }}{q}\ln \left( {z_{2} + \sqrt {1 + z_{2}^{2} } } \right) - \frac{{H_{2}^{2} z_{3} }}{{EAq}} - \frac{{H_{2} }}{q}\ln \left( {z_{3} + \sqrt {1 + z_{3}^{2} } } \right) + \hfill \\ R_{1} \left( {\sin \arctan z_{2} - \sin \gamma _{1} } \right) + R_{2} \left( {\sin \gamma _{3} - \sin \arctan z_{3} } \right) \hfill \\ = L_{2} + \Delta l_{1} \cos \varphi _{1} - \Delta x_{1} \hfill \\ \end{gathered}$$

9 $$\frac{{H_{2} }}{q}\left( {z_{2} - z_{3} } \right) + R_{1} \left( {\arctan z_{2} - \gamma_{1} } \right) + R_{2} \left( {\gamma_{3} - \arctan z_{3} } \right) = S_{2}$$

for the right side span:

10 $$\begin{gathered} \frac{{H_{4}^{2} z_{7}^{2} }}{{2EAq}} + \frac{{H_{4} }}{q}\sqrt {1 + z_{7}^{2} } - \frac{{H_{4}^{2} z_{8}^{2} }}{{2EAq}} - \frac{{H_{4} }}{q}\sqrt {1 + z_{8}^{2} } + \hfill \\ R_{1} \left( {\cos \gamma _{2} - \cos \arctan z_{7} } \right) + R_{2} \left( {\cos \arctan z_{8} - \cos \gamma _{4} } \right) \hfill \\ = h_{3} + \Delta l_{2} \sin \varphi _{2} \hfill \\ \end{gathered}$$

11 $$\begin{gathered} \frac{{H_{4}^{2} z_{7} }}{{EAq}} + \frac{{H_{4} }}{q}\ln \left( {z_{7} + \sqrt {1 + z_{7}^{2} } } \right) - \frac{{H_{4}^{2} z_{8} }}{{EAq}} - \frac{{H_{4} }}{q}\ln \left( {z_{8} + \sqrt {1 + z_{8}^{2} } } \right) + \hfill \\ R_{1} \left( {\sin \arctan z_{7} - \sin \gamma _{2} } \right) + R_{2} \left( {\sin \gamma _{4} - \sin \arctan z_{8} } \right) \hfill \\ = L_{3} + \Delta l_{2} \cos \varphi _{2} - \Delta x_{2} \hfill \\ \end{gathered}$$

12 $$\frac{{H_{4} }}{q}\left( {z_{7} - z_{8} } \right) + R_{1} \left( {\arctan z_{7} - \gamma_{2} } \right) + R_{2} \left( {\gamma_{4} - \arctan z_{8} } \right) = S_{3}$$

for the left anchor span:

13 $$\begin{aligned} \frac{{H_{3}^{2} z_{4}^{2} }}{{2EAq}} + & \frac{{H_{3} }}{q}\sqrt {1 + z_{4}^{2} } - \frac{{H_{3}^{2} z_{5}^{2} }}{{2EAq}} - \frac{{H_{3} }}{q}\sqrt {1 + z_{5}^{2} } \\ + & R_{2} \left( {\cos \gamma _{3} - \cos \arctan z_{4} } \right) = h_{4} - \Delta l_{1} \sin \varphi _{1} \\ \end{aligned}$$

14 $$\begin{aligned} \frac{{H_{3}^{2} z_{4} }}{{EAq}} + & \frac{{H_{3} }}{q}\ln \left( {z_{4} + \sqrt {1 + z_{4}^{2} } } \right) - \frac{{H_{3}^{2} z_{5} }}{{EAq}} - \frac{{H_{3} }}{q}\ln \left( {z_{5} + \sqrt {1 + z_{5}^{2} } } \right) \\ + & R_{2} \left( {\sin \gamma _{3} - \sin \arctan z_{4} } \right) = L_{4} - \Delta l_{1} \cos \varphi _{1} \\ \end{aligned}$$

15 $$\frac{{H_{3} }}{q}\left( {z_{4} - z_{5} } \right) + R_{2} \left( {\arctan z_{4} - \gamma_{3} } \right) = S_{4}$$

and for the right anchor span:

16 $$\begin{aligned} \frac{{H_{5}^{2} z_{9}^{2} }}{{2EAq}} + & \frac{{H_{5} }}{q}\sqrt {1 + z_{9}^{2} } - \frac{{H_{5}^{2} z_{{10}}^{2} }}{{2EAq}} - \frac{{H_{5} }}{q}\sqrt {1 + z_{{10}}^{2} } \\ + & R_{2} \left( {\cos \gamma _{4} - \cos \arctan z_{9} } \right) = h_{5} - \Delta l_{2} \sin \varphi _{2} \\ \end{aligned}$$

17 $$\begin{aligned} \frac{{H_{5}^{2} z_{9} }}{{EAq}} + & \frac{{H_{5} }}{q}\ln \left( {z_{9} + \sqrt {1 + z_{9}^{2} } } \right) - \frac{{H_{5}^{2} z_{{10}} }}{{EAq}} - \frac{{H_{5} }}{q}\ln \left( {z_{{10}} + \sqrt {1 + z_{{10}}^{2} } } \right) \\ + & R_{2} \left( {\sin \gamma _{4} - \sin \arctan z_{9} } \right) = L_{5} - \Delta l_{2} \cos \varphi _{2} \\ \end{aligned}$$

18 $$\frac{{H_{5} }}{q}\left( {z_{9} - z_{10} } \right) + R_{2} \left( {\arctan z_{9} - \gamma_{4} } \right) = S_{5}$$

The equilibrium condition of the tower and splay saddles is as follows:

for the left side of the whole bridge:

19 $$H_{1} = H_{2}$$

20 $$H_{2} \left( {\cos \varphi_{1} + z_{3} \sin \varphi_{1} } \right) = H_{3} \left( {\cos \varphi_{1} + z_{4} \sin \varphi_{1} } \right)$$

for the right side of the whole bridge:

21 $$H_{1} = H_{4}$$

22 $$H_{4} \left( {\cos \varphi_{2} + z_{8} \sin \varphi_{2} } \right) = H_{5} \left( {\cos \varphi_{2} + z_{9} \sin \varphi_{2} } \right)$$

Note that the geometrical relationship between the above main cable and the saddles may vary with the radius of the saddles. When programming, selection and judgment can be made according to the relationship between the obtained cable saddle tangent point and the circle centre. The calculation of the cable saddle pre-offsets requires solving several coupled non-linear equations composed of Eqs. (4–22) and reaching up to 19. The simultaneous equations can then be worked out by the Newton–Raphson method.

## Calculation of cable saddle pre-offset equations using the Newton–Raphson method

### Newton–Raphson method for solving cable saddle pre-offsets equations

Rewrite Eq. (4) to (22) as follows:

23 $$\begin{gathered} f_{i} \left( {x_{1} ,x_{2} , \cdots ,x_{19} } \right) \hfill \\ = f_{i} \left( {z_{1} ,z_{2} ,z_{3} ,z_{4} ,z_{5} ,z_{6} ,z_{7} ,z_{8} ,z_{9} ,z_{10} ,H_{1} ,H_{2} ,H_{3} ,H_{4} ,H_{5} ,\Delta x_{1} ,\Delta x_{2} ,\Delta l_{1} ,\Delta l_{2} } \right) \hfill \\ = 0\left( {i = 1,2, \cdots ,19} \right) \hfill \\ \end{gathered}$$

The determination variables are set as follows:

24 $$X{ = }\left\{ {x_{1} ,x_{2} , \cdots ,x_{i} } \right\}^{T} = \left\{ {x_{v} } \right\}$$

where *x*_*v*_ is the component of vector *X* and *j* is the number of variables.

The dependent variables are set as follows:

25 $$F = \left\{ {f_{1} ,f_{2} , \cdots ,f_{i} } \right\}^{T} = \left\{ {f_{v} } \right\}$$

where *f*_*v*_ is the component of vector *F* and *i* is the number of dependent variables.

In a continuous neighbourhood of *X*, expand the Taylor series of *f*_*i*_ as:

26 $$f_{i} \left( {X + \delta X} \right) = f_{i} \left( X \right) + \sum\limits_{j = 1}^{19} {\frac{{\partial f_{i} }}{{\partial x_{j} }}\delta x_{j} + o\left( {\delta X^{2} } \right) = 0}$$

where $o\left( {\delta X^{2} } \right)$ is a higher-order infinitesimal, which can be neglected.

The derivative of *f*_*i*_ with respect to *x*_*j*_ is can be expressed as follows:

27 $$J_{ij} = \frac{{\partial f_{i} }}{{\partial x_{j} }}$$

The Jacobi matrix *J* is defined as follows:

28 $$J{ = }\left\{ {J_{ij} } \right\}$$

The iterative formulas are expressed as follows:

29 $$\delta X^{k} = \frac{{ - F^{k} }}{{J^{K} }}$$

30 $$X^{k} = X^{{k{ - }1}} + \delta X^{k}$$

where *k* is the number of iterations.

The programming calculation process in this paper is as follows:

1. Select the initial $X^{0}$
  $$X^{0} { = }(x_{1}^{0} ,x_{2}^{0} , \ldots ,x_{19}^{0} ) = (z_{1}^{0} ,z_{2}^{0} , \cdots ,z_{10}^{0} ,H_{1}^{0} ,H_{2}^{0} ,H_{3}^{0} ,H_{4}^{0} ,H_{5}^{0} ,\Delta x_{1}^{0} ,\Delta x_{2}^{0} ,\Delta l_{1}^{0} ,\Delta l_{2}^{0} );$$

set the solution accuracy $\varepsilon$ and assign a value of 1 to the iterative times *k*.

(2) Calculate $$D_{i} = - f_{i} \left( {X^{k - 1} } \right)\left( {i = 1,2, \cdots ,19} \right)$$

If “$\max \left| {D_{i} } \right| \le \varepsilon$” is true and the constraints are satisfied, then the solution of the equations is as follows:

$$X^{k - 1} = (x_{1}^{k - 1} ,x_{2}^{k - 1} , \ldots ,x_{19}^{k - 1} ) = (z_{1}^{k - 1} ,z_{2}^{k - 1} , \cdots ,z_{10}^{k - 1} ,H_{1}^{k - 1} , \cdots ,H_{5}^{k - 1} ,\Delta x_{1}^{k - 1} ,\Delta x_{2}^{k - 1} ,\Delta l_{1}^{k - 1} ,\Delta l_{2}^{k - 1} )$$

end.

If the above conditions are not satisfied, go to the third step.

(3) Obtain the Jacobi matrix $J_{ij}$, $\delta X^{k}$, and $X^{k}$ by using Eqs. (27), (29), and (30), respectively. The iteration is not finished until the stopping criterion is satisfied.

**Figure 7:**
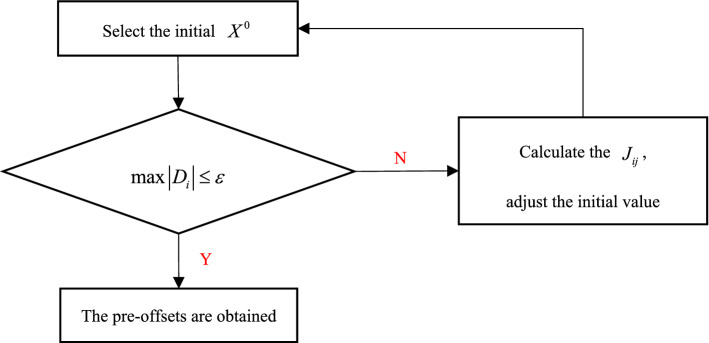
Algorithm flow of the tower and splay saddle pre-offsets.

The algorithm flow is shown in Fig. 7.

In this way, the pre-offsets of the tower and splay saddle in the unloaded state are obtained, and the configuration and the internal force of the main cable are simultaneously obtained by the above calculation process.

### Derivation of the Jacobi matrix

For Eqs. (4)–(22), define the matrix function *F* as:

31 $$\begin{aligned} F_{1} & = \frac{{H_{1}^{2} z_{1} }}{{EAq}} + \frac{{H_{1} }}{q}\ln \left( {z_{1} + \sqrt {1 + z_{1}^{2} } } \right) + R_{1} \left( {\sin \gamma _{1} + \sin \arctan z_{1} } \right) \\ & \quad \quad {\text{ + }}\frac{{H_{1}^{2} z_{6} }}{{EAq}} + \frac{{H_{1} }}{q}\ln \left( {z_{6} + \sqrt {1 + z_{6}^{2} } } \right) + R_{1} \left( {\sin \gamma _{2} + \sin \arctan z_{6} } \right) - L_{1} - \Delta x_{1} - \Delta x_{2} \\ \end{aligned}$$

32 $$F_{2} = \frac{{H_{1} z_{1} }}{q} + R_{1} \left( {\gamma_{1} + \arctan z_{1} } \right){ + }\frac{{H_{1} z_{6} }}{q} + R_{1} \left( {\gamma_{2} + \arctan z_{6} } \right) - S_{1}$$

33 $$\begin{aligned} F_{3} = & \frac{{H_{1}^{2} z_{6}^{2} }}{{2EAq}} + \frac{{H_{1} }}{q}\sqrt {1 + z_{6}^{2} } - \frac{{H_{1}^{2} z_{1}^{2} }}{{2EAq}} - \frac{{H_{1} }}{q}\sqrt {1 + z_{1}^{2} } \\ \quad + & R_{1} \left( {\cos \gamma _{2} - \cos \arctan z_{6} } \right) - R_{1} \left( {\cos \gamma _{1} - \cos \arctan z_{1} } \right) \\ \quad - & h_{1} \\ \end{aligned}$$

34 $$\begin{aligned} F_{4} = & \frac{{H_{2}^{2} z_{2}^{2} }}{{2EAq}} + \frac{{H_{2} }}{q}\sqrt {1 + z_{2}^{2} } - \frac{{H_{2}^{2} z_{3}^{2} }}{{2EAq}} - \frac{{H_{2} }}{q}\sqrt {1 + z_{3}^{2} } \\ \quad + & R_{1} \left( {\cos \gamma _{1} - \cos \arctan z_{2} } \right) + R_{2} \left( {\cos \arctan z_{3} - \cos \gamma _{3} } \right) \\ \quad - & h_{2} - \Delta l_{1} \sin \varphi _{1} \\ \end{aligned}$$

35 $$\begin{aligned} F_{5} = & \frac{{H_{2}^{2} z_{2} }}{{EAq}} + \frac{{H_{2} }}{q}\ln \left( {z_{2} + \sqrt {1 + z_{2}^{2} } } \right) - \frac{{H_{2}^{2} z_{3} }}{{EAq}} - \frac{{H_{2} }}{q}\ln \left( {z_{3} + \sqrt {1 + z_{3}^{2} } } \right) \\ \quad + & R_{1} \left( {\sin \arctan z_{2} - \sin \gamma _{1} } \right) + R_{2} \left( {\sin \gamma _{3} - \sin \arctan z_{3} } \right) \\ \quad - & L_{2} - \Delta l_{1} \cos \varphi _{1} + \Delta x_{1} \\ \end{aligned}$$

36 $$F_{6} = \frac{{H_{2} }}{q}\left( {z_{2} - z_{3} } \right) + R_{1} \left( {\arctan z_{2} - \gamma_{1} } \right) + R_{2} \left( {\gamma_{3} - \arctan z_{3} } \right) - S_{2}$$

37 $$\begin{aligned} F_{7} = & \frac{{H_{4}^{2} z_{7}^{2} }}{{2EAq}} + \frac{{H_{4} }}{q}\sqrt {1 + z_{7}^{2} } - \frac{{H_{4}^{2} z_{8}^{2} }}{{2EAq}} - \frac{{H_{4} }}{q}\sqrt {1 + z_{8}^{2} } \\ \quad + & R_{1} \left( {\cos \gamma _{2} - \cos \arctan z_{7} } \right) + R_{2} \left( {\cos \arctan z_{8} - \cos \gamma _{4} } \right) \\ \quad - & h_{3} - \Delta l_{2} \sin \varphi _{2} \\ \end{aligned}$$

38 $$\begin{aligned} F_{8} = & \frac{{H_{4}^{2} z_{7} }}{{EAq}} + \frac{{H_{4} }}{q}\ln \left( {z_{7} + \sqrt {1 + z_{7}^{2} } } \right) - \frac{{H_{4}^{2} z_{8} }}{{EAq}} - \frac{{H_{4} }}{q}\ln \left( {z_{8} + \sqrt {1 + z_{8}^{2} } } \right) \\ \quad + & R_{1} \left( {\sin \arctan z_{7} - \sin \gamma _{2} } \right) + R_{2} \left( {\sin \gamma _{4} - \sin \arctan z_{8} } \right) \\ \quad - & L_{3} - \Delta l_{2} \cos \varphi _{2} + \Delta x_{2} \\ \end{aligned}$$

39 $$F_{9} = \frac{{H_{4} }}{q}\left( {z_{7} - z_{8} } \right) + R_{1} \left( {\arctan z_{7} - \gamma_{2} } \right) + R_{2} \left( {\gamma_{4} - \arctan z_{8} } \right) - S_{3}$$

40 $$\begin{aligned} F_{{10}} = & \frac{{H_{3}^{2} z_{4}^{2} }}{{2EAq}} + \frac{{H_{3} }}{q}\sqrt {1 + z_{4}^{2} } - \frac{{H_{3}^{2} z_{5}^{2} }}{{2EAq}} - \frac{{H_{3} }}{q}\sqrt {1 + z_{5}^{2} } \\ \quad + & R_{2} \left( {\cos \gamma _{3} - \cos \arctan z_{4} } \right) - h_{4} + \Delta l_{1} \sin \varphi _{1} \\ \end{aligned}$$

41 $$\begin{gathered} F_{11} = \frac{{H_{3}^{2} z_{4} }}{EAq} + \frac{{H_{3} }}{q}\ln \left( {z_{4} + \sqrt {1 + z_{4}^{2} } } \right) - \frac{{H_{3}^{2} z_{5} }}{EAq} - \frac{{H_{3} }}{q}\ln \left( {z_{5} + \sqrt {1 + z_{5}^{2} } } \right) \hfill \\ \quad + R_{2} \left( {\sin \gamma_{3} - \sin \arctan z_{4} } \right) - L_{4} + \Delta l_{1} \cos \varphi_{1} \hfill \\ \end{gathered}$$

42 $$F_{12} = \frac{{H_{3} }}{q}\left( {z_{4} - z_{5} } \right) + R_{2} \left( {\arctan z_{4} - \gamma_{3} } \right) - S_{4}$$

43 $$\begin{aligned} F_{{13}} = & \frac{{H_{5}^{2} z_{9}^{2} }}{{2EAq}} + \frac{{H_{5} }}{q}\sqrt {1 + z_{9}^{2} } - \frac{{H_{5}^{2} z_{{10}}^{2} }}{{2EAq}} - \frac{{H_{5} }}{q}\sqrt {1 + z_{{10}}^{2} } \\ \quad + & R_{2} \left( {\cos \gamma _{4} - \cos \arctan z_{9} } \right) - h_{5} + \Delta l_{2} \sin \varphi _{2} \\ \end{aligned}$$

44 $$\begin{aligned} F_{{14}} = & \frac{{H_{5}^{2} z_{9} }}{{EAq}} + \frac{{H_{5} }}{q}\ln \left( {z_{9} + \sqrt {1 + z_{9}^{2} } } \right) - \frac{{H_{5}^{2} z_{{10}} }}{{EAq}} - \frac{{H_{5} }}{q}\ln \left( {z_{{10}} + \sqrt {1 + z_{{10}}^{2} } } \right) \\ \quad + & R_{2} \left( {\sin \gamma _{4} - \sin \arctan z_{9} } \right) - L_{5} + \Delta l_{2} \cos \varphi _{2} \\ \end{aligned}$$

45 $$F_{15} = \frac{{H_{5} }}{q}\left( {z_{9} - z_{10} } \right) + R_{2} \left( {\arctan z_{9} - \gamma_{4} } \right) - S_{5}$$

46 $$F_{16} = H_{1} - H_{2}$$

47 $$F_{17} = H_{2} \left( {\cos \varphi_{1} + z_{3} \sin \varphi_{1} } \right) - H_{3} \left( {\cos \varphi_{1} + z_{4} \sin \varphi_{1} } \right)$$

48 $$F_{18} = H_{1} - H_{4}$$

49 $$F_{19} = H_{4} \left( {\cos \varphi_{2} + z_{8} \sin \varphi_{2} } \right) - H_{5} \left( {\cos \varphi_{2} + z_{9} \sin \varphi_{2} } \right)$$

Substituting these into the Eq. (27) leads to the Jacobi matrix:

50 $$J = \left[ {J_{ij} } \right]_{19 \times 19}$$

where

51 $$\begin{aligned} J_{{1j}} & = \left[ {\frac{{H_{1}^{2} }}{{EAq}} + \frac{{H_{1} }}{q}\frac{1}{{\sqrt {1 + z_{1}^{2} } }} + \frac{{R_{1} }}{{\left( {1 + z_{1}^{2} } \right)^{{\frac{3}{2}}} }},0,0,0,0,\frac{{H_{1}^{2} }}{{EAq}} + \frac{{H_{1} }}{q}\frac{1}{{\sqrt {1 + z_{6}^{2} } }} + \frac{{R_{1} }}{{\left( {1 + z_{6}^{2} } \right)^{{\frac{3}{2}}} }},0,0,0,0,} \right. \\ & \quad \quad \left. {\frac{{2H_{1} z_{1} }}{{EAq}} + \frac{{\ln \left( {z_{1} + \sqrt {1 + z_{1}^{2} } } \right)}}{q} + \frac{{2H_{1} z_{6} }}{{EAq}} + \frac{{\ln \left( {z_{6} + \sqrt {1 + z_{6}^{2} } } \right)}}{q},0,0,0,0, - 1, - 1,0,0} \right] \\ \end{aligned}$$

52 $$J_{2j} = \left[ {\frac{{H_{1} }}{q} + \frac{{R_{1} }}{{\sqrt {1 + z_{1}^{2} } }},0,0,0,0,\frac{{H_{1} }}{q} + \frac{{R_{1} }}{{\sqrt {1 + z_{6}^{2} } }},0,0,0,0,\frac{{z_{1} }}{q} + \frac{{z_{6} }}{q},0,0,0,0,0,0,0,0} \right]$$

53 $$\begin{aligned} J_{{3j}} & = \left[ { - \frac{{H_{1}^{2} z_{1} }}{{EAq}} + \frac{{H_{1} }}{q}\frac{{z_{1} }}{{\sqrt {1 + z_{1}^{2} } }} + R_{1} \frac{{z_{1} }}{{\left( {1 + z_{1}^{2} } \right)^{{\frac{3}{2}}} }},0,0,0,0,\frac{{H_{1}^{2} z_{6} }}{{EAq}} + \frac{{H_{1} }}{q}\frac{{z_{6} }}{{\sqrt {1 + z_{6}^{2} } }}} \right. \\ & \quad \quad \left. { - R_{1} \frac{{z_{6} }}{{\left( {1 + z_{6}^{2} } \right)^{{\frac{3}{2}}} }},0,0,0,0,\frac{{H_{1} z_{6}^{2} }}{{EAq}} + \frac{{\sqrt {1 + z_{6}^{2} } }}{q} - \frac{{H_{1} z_{1}^{2} }}{{EAq}} - \frac{{\sqrt {1 + z_{1}^{2} } }}{q},0,0,0,0,0,0,0,0} \right] \\ \end{aligned}$$

54 $$\begin{aligned} J_{{4j}} & = \left[ {0,\frac{{H_{2}^{2} z_{2} }}{{EAq}} + \frac{{H_{2} }}{q}\frac{{z_{2} }}{{\sqrt {1 + z_{2}^{2} } }} - R_{1} \frac{{z_{2} }}{{\left( {1 + z_{2}^{2} } \right)^{{\frac{3}{2}}} }}, - \frac{{H_{2}^{2} z_{3} }}{{EAq}} - \frac{{H_{2} }}{q}\frac{{z_{3} }}{{\sqrt {1 + z_{3}^{2} } }} + R_{2} \frac{{z_{3} }}{{\left( {1 + z_{3}^{2} } \right)^{{\frac{3}{2}}} }}} \right. \\ & \quad \quad \left. {0,0,0,0,0,0,0,0,\frac{{H_{2} z_{2}^{2} }}{{EAq}} + \frac{{\sqrt {1 + z_{2}^{2} } }}{q} - \frac{{H_{2} z_{3}^{2} }}{{EAq}} - \frac{{\sqrt {1 + z_{3}^{2} } }}{q},0,0,0,0,0,0, - \sin \varphi _{1} ,0} \right] \\ \end{aligned}$$

55 $$\begin{aligned} J_{{5j}} & = \left[ {0,\frac{{H_{2}^{2} }}{{EAq}} + \frac{{H_{2} }}{q}\frac{1}{{\sqrt {1 + z_{2}^{2} } }} + R_{1} \frac{1}{{\left( {1 + z_{2}^{2} } \right)^{{3/2}} }}, - \frac{{H_{2}^{2} }}{{EAq}} - \frac{{H_{2} }}{q}\frac{1}{{\sqrt {1 + z_{3}^{2} } }} - R_{2} \frac{1}{{\left( {1 + z_{3}^{2} } \right)^{{3/2}} }},0,0,0,0,} \right.[ \\ & \quad \quad \left. {0,0,0,\frac{{2H_{2} z_{2} }}{{EAq}} + \frac{{\ln \left( {z_{2} + \sqrt {1 + z_{2}^{2} } } \right)}}{q} - \frac{{2H_{2} z_{3} }}{{EAq}} - \frac{{\ln \left( {z_{3} + \sqrt {1 + z_{3}^{2} } } \right)}}{q},0,0,0,1,0, - \cos \varphi _{1} ,0} \right] \\ \end{aligned}$$

56 $$J_{6j} = \left[ {0,\frac{{H_{2} }}{q} + \frac{{R_{1} }}{{1 + z_{2}^{2} }}, - (\frac{{H_{2} }}{q} + \frac{{R_{2} }}{{1 + z_{3}^{2} }}),0,0,0,0,0,0,0,0,\frac{1}{q}\left( {z_{2} - z_{3} } \right),0,0,0,0,0,0,0} \right]$$

57 $$\begin{aligned} J_{{7j}} & = \left[ {0,0,0,0,0,0,0,\frac{{H_{4}^{2} z_{7} }}{{EAq}} + \frac{{H_{4} }}{q}\frac{{z_{7} }}{{\sqrt {1 + z_{7}^{2} } }} - R_{1} \frac{{z_{7} }}{{\left( {1 + z_{7}^{2} } \right)^{{\frac{3}{2}}} }}, - \frac{{H_{4}^{2} z_{8} }}{{EAq}} - \frac{{H_{4} }}{q}\frac{{z_{8} }}{{\sqrt {1 + z_{8}^{2} } }} + R_{2} \frac{{z_{8} }}{{\left( {1 + z_{8}^{2} } \right)^{{\frac{3}{2}}} }},} \right. \\ & \quad \quad \left. {0,0,0,0,0,\frac{{H_{4} z_{7}^{2} }}{{EAq}} + \frac{{\sqrt {1 + z_{7}^{2} } }}{q} - \frac{{H_{2} z_{8}^{2} }}{{EAq}} - \frac{{\sqrt {1 + z_{8}^{2} } }}{q},0,0,0,0, - \sin \varphi _{2} } \right] \\ \end{aligned}$$

58 $$\begin{aligned} J_{{8j}} & = \left[ {0,0,0,0,0,0,\frac{{H_{4}^{2} }}{{EAq}} + \frac{{H_{4} }}{q}\frac{1}{{\sqrt {1 + z_{7}^{2} } }} + R_{1} \frac{1}{{\left( {1 + z_{7}^{2} } \right)^{{3/2}} }}, - \frac{{H_{4}^{2} }}{{EAq}} - \frac{{H_{4} }}{q}\frac{1}{{\sqrt {1 + z_{8}^{2} } }} - R_{2} \frac{1}{{\left( {1 + z_{8}^{2} } \right)^{{3/2}} }},} \right. \\ & \quad \quad \left. {0,0,0,0,0,\frac{{2H_{4} z_{7} }}{{EAq}} + \frac{{\ln \left( {z_{7} + \sqrt {1 + z_{7}^{2} } } \right)}}{q} - \frac{{2H_{7} z_{8} }}{{EAq}} - \frac{{\ln \left( {z_{8} + \sqrt {1 + z_{8}^{2} } } \right)}}{q},0,0,1,0, - \cos \varphi _{2} } \right] \\ \end{aligned}$$

59 $$J_{9j} = \left[ {0,0,0,0,0,0,\frac{{H_{4} }}{q} + \frac{{R_{1} }}{{1 + z_{7}^{2} }}, - (\frac{{H_{4} }}{q} + \frac{{R_{2} }}{{1 + z_{8}^{2} }}),0,0,0,0,0,\frac{1}{q}\left( {z_{7} - z_{8} } \right),0,0,0,0,0} \right]$$

60 $$\begin{aligned} J_{{10j}} & = \left[ {0,0,0,\frac{{H_{3}^{2} z_{4} }}{{EAq}} + \frac{{H_{3} z_{4} }}{{q\sqrt {1 + z_{4}^{2} } }} - R_{2} \frac{{z_{4} }}{{\left( {1 + z_{4}^{2} } \right)^{{3/2}} }}, - \frac{{H_{3}^{2} z_{5} }}{{EAq}} - \frac{{H_{3} z_{5} }}{{q\sqrt {1 + z_{5}^{2} } }},0,0,0,0,0,} \right. \\ & \quad \quad \left. {0,0,\frac{{H_{3} z_{4}^{2} }}{{EAq}} + \frac{{\sqrt {1 + z_{4}^{2} } }}{q} - \frac{{H_{3} z_{5}^{2} }}{{EAq}} - \frac{{\sqrt {1 + z_{5}^{2} } }}{q},0,0,0,0,0,\sin \varphi _{1} ,0} \right] \\ \end{aligned}$$

61 $$\begin{aligned} J_{{11j}} & = \left[ {0,0,0,\frac{{H_{3}^{2} }}{{EAq}} + \frac{{H_{3} }}{{q\sqrt {1 + z_{4}^{2} } }} - R_{2} \frac{1}{{\left( {1 + z_{4}^{2} } \right)^{{3/2}} }}, - \frac{{H_{3}^{2} }}{{EAq}} - \frac{{H_{3} }}{{q\sqrt {1 + z_{5}^{2} } }},0,0,0,0,0,0,0,} \right. \\ & \quad \quad \left. {\frac{{2H_{3} z_{4} }}{{EAq}} + \frac{1}{q}\ln (z_{4} + \sqrt {1 + z_{4}^{2} } ) - \frac{{2H_{3} z_{5} }}{{EAq}} - \frac{1}{q}\ln (z_{5} + \sqrt {1 + z_{5}^{2} } ),0,0,0,0,\cos \varphi _{1} ,0} \right] \\ \end{aligned}$$

62 $$J_{12j} = \left[ {0,0,0,\frac{{H_{3} }}{q} + \frac{{R_{2} }}{{1 + z_{4}^{2} }}, - \frac{{H_{3} }}{q},0,0,0,0,0,0,0,\frac{1}{q}\left( {z_{4} - z_{5} } \right),0,0,0,0,0,0} \right]$$

63 $$\begin{aligned} J_{{13j}} &= \left[ {0,0,0,0,0,0,0,0,\frac{{H_{5}^{2} z_{9} }}{{EAq}} + \frac{{H_{5} z_{9} }}{{q\sqrt {1 + z_{9}^{2} } }} - R_{2} \frac{{z_{9} }}{{\left( {1 + z_{9}^{2} } \right)^{{3/2}} }}, - \frac{{H_{5}^{2} z_{{10}} }}{{EAq}} - \frac{{H_{5} z_{{10}} }}{{q\sqrt {1 + z_{{10}}^{2} } }},0,0,0,0,} \right. \\ & \quad \left. {\frac{{H_{5} z_{9}^{2} }}{{EAq}} + \frac{{\sqrt {1 + z_{9}^{2} } }}{q} - \frac{{H_{3} z_{{10}}^{2} }}{{EAq}} - \frac{{\sqrt {1 + z_{{10}}^{2} } }}{q},0,0,0,\sin \varphi _{2} } \right] \\ \end{aligned}$$

64 $$\begin{aligned} J_{{14j}} & = \left[ {0,0,0,0,0,0,0,0,\frac{{H_{5}^{2} }}{{EAq}}{\text{ + }}\frac{{H_{5} }}{{q\sqrt {1 + z_{9}^{2} } }} - R_{2} \frac{1}{{\left( {1 + z_{9}^{2} } \right)^{{3/2}} }}, - \frac{{H_{5}^{2} }}{{EAq}} - \frac{{H_{5} }}{{q\sqrt {1 + z_{{10}}^{2} } }},} \right. \\ & \quad \quad \left. {0,0,0,0,\frac{{2H_{5} z_{9} }}{{EAq}} + \frac{1}{q}\ln (z_{9} + \sqrt {1 + z_{9}^{2} } ) - \frac{{2H_{5} z_{{10}} }}{{EAq}} - \frac{1}{q}\ln (z_{{10}} + \sqrt {1 + z_{{10}}^{2} } ),0,0,0,\cos \varphi _{2} } \right] \\ \end{aligned}$$

65 $$J_{15j} = \left[ {0,0,0,0,0,0,0,0,\frac{{H_{5} }}{q} + \frac{{R_{2} }}{{1 + z_{9}^{2} }}, - \frac{{H_{5} }}{q},0,0,0,0,\frac{1}{q}\left( {z_{9} - z_{10} } \right),0,0,0,0} \right]$$

66 $$J_{16j} = [0,0,0,0,0,0,0,0,0,0,1, - 1,0,0,0,0,0,0,0]$$

67 $$\begin{aligned} J_{{17j}} & = \left[ {0,0,H_{2} \sin \varphi _{1} , - H_{3} \sin \varphi _{1} ,0,0,0,0,0,0,0,} \right. \\ & \quad \quad \left. {\cos \varphi _{1} + z_{3} \sin \varphi _{1} , - \left( {\cos \varphi _{1} + z_{4} \sin \varphi _{1} } \right),0,0,0,0,0,0} \right] \\ \end{aligned}$$

68 $$J_{18j} = [0,0,0,0,0,0,0,0,0,0,1,0,0, - 1,0,0,0,0,0]$$

69 $$\begin{aligned} J_{{19j}} & = \left[ {0,0,0,0,0,0,0,H_{4} \sin \varphi _{2} , - H_{5} \sin \varphi _{2} ,0,0,} \right. \\ & \quad \quad \left. {0,0,\cos \varphi _{2} + z_{8} \sin \varphi _{2} , - \left( {\cos \varphi _{2} + z_{9} \sin \varphi _{2} } \right),0,0,0,0} \right]. \\ \end{aligned}$$

### Selection principle of the initial value *X*_0_

To solve the nonlinear equations with the Newton–Raphson algorithm it is essential to select iterative initial values. Moreover, the iterative process may not even converge if the initial values are far from the actual results.

In this paper, the method of selecting the initial values is as follows:

According to the position of the cable saddles in the completed state of the bridge, the main cable configuration and the inner force are obtained. Consider the slopes of the tangent point of the tower and splay saddles and the anchor point as the initial values of *z*_1_, *z*_2_, *z*_3_, *z*_4_, *z*_5_, *z*_6_, *z*_7_, *z*_8_, *z*_9_, and *z*_10_, respectively, and the horizon tension of the main cable in the cable segments as the initial values of *H*_1_, *H*_2_,*H*_3_, *H*_4_, and *H*_5_, respectively. The initial value of the tower saddle and the splay saddles offset along the sliding surface can be taken as 1 m and 0.1 m, respectively. Then, the homotopy continuation method is applied to obtain the best initial values to reduce the influence of the initial value selection on the convergence of the Newton–Raphson algorithm.

Therefore, the function *H(X,s)* can be expressed as follows:

70 $$H\left( {X,s} \right) = H_{i} \left( {x_{1} ,x_{2} , \cdots ,x_{10} ,s} \right) = sF\left( X \right) + \left( {1 - s} \right)F_{0} \left( X \right)\left( {i = 1,2, \cdots ,19} \right)$$

where the value range of *s* is [0,1] and *F(X)* is the homotopy to the primitive function *F(X*_0_*)*.

71 $$F_{0} \left( X \right) = F\left( X \right) - F\left( {X_{0} } \right),$$

where *X*_0_ is the selected initial value. Substituting Eq. (71) into Eq. (70), then

72 $$H\left( {X,s} \right) = H_{i} \left( {x_{1} ,x_{2} , \cdots ,x_{19} ,s} \right) = F\left( X \right) + \left( {s - 1} \right)F\left( {X_{0} } \right)\left( {i = 1,2, \cdots ,19} \right)$$

In this case, *H(X,s)* has the same derivative as *F(X)*. The Newton–Raphson iterative process can generally ensure that the equations converge quickly under the condition of replacing *F(X)* with *H(X,s)*.

## Analytical verification

The area of the cross section of a suspension bridge main cable *A* = 1.488 m^2^, the distributed load of the main cable *q* = 116.03 kN/m, and the elastic modulus *E* = 200,000 MPa. The radii of the tower and splay saddles are 10.57 m and 7.5 m, respectively. In the completed state of the bridge, the horizon distance and the vertical distance between the fixed points of the main cable saddle on the left and right sides of the main span *L*_1_ = 1649.9729 m and *h*_1_ = 120.3 m; the unstrained length of the above section *S*_1_ = 1671.3033 m; the angle between the line connecting the fixed point of the main cable saddle with the centre of the circle and the perpendicular line passing through the centre of the circle *γ*_1_ = 0.02418446 rad and *γ*_2_ = 0.030656345 rad; the horizontal distance and the vertical distance from the fixed point of the main cable saddle of the side span to the fixed point of the splay saddle *L*_2_ = 465.6 m, *h*_2_ = 220.3 m, *L*_3_ = 465.6 m and *h*_3_ = 350.6 m, respectively; the unstrained length of the above section *S*_2_ = 513.8171 m and *S*_3_ = 582.3618 m; the angle between the line connecting the fixed point of the splay saddle with the centre of the circle and the perpendicular line passing through the centre of the circle *γ*_3_ = 0.49318446 rad and *γ*_4_ = 0.540656345 rad; the horizon distance and the perpendicular distance from the fixed point of the splay saddle of the anchor span to the anchor point *L*_4_ = 25.2795 m, *h*_4_ = 21.2120 m, *L*_5_ = 25.2795 m and *h*_5_ = 21.2120 m, respectively; the unstrained length of the above segment *S*_4_ = 33.4 m and *S*_5_ = 32.4 m, and the angle of the sliding surface of the splay saddle *φ*_1_ = 33.6784° and *φ*_2_ = 37.6911°.

The main cable configuration and inner force are obtained according to the position of the cable saddles in the completed situation of the bridge, including:

*H*_1_ = 362,580.4kN; *H*_2_ = 794,781.1 kN; *H*_3_ = 3,529,202.9 kN; *H*_4_ = 470,497.7 kN; *H*_5_ = 6,478,413.5 kN; *z*_1_ = 0.1920; *z*_2_ = 0.5127; *z*_3_ = 0.4386; *z*_4_ = 0.7976; *z*_5_ = 0.7965; *z*_6_ = 0.3411; *z*_7_ = 0.8305; *z*_8_ = 0.6887; *z*_9_ = 0.8269; *z*_10_ = 0.8264.

Considering the above values as the iterative initial values, the initial values Δ*x*_*i*_ (*i* = 1*,*2) and Δ*l*_*i*_(*i* = 1,2) are taken as 1 m and 0.1 m, respectively. Then, the calculated results in the program are shown in Table 1.The iterative results are listed in Table 2 adopting the method of Ref.^16^.

**Table 1:** Results of cable saddles pre-offsets adopting the method of this paper.

Offsets	Calculation results (left/right)
Offset of the tower saddles/m	2.3683/2.4242
Offset of the splay saddles/m	1.2582/1.8132
Horizon tension of the main cable on the main span side of the tower saddle in the unload state after pre-offsetting/kN	416291.9/416291.9
Horizon tension of the main cable on the side span side of the tower saddle in the unloaded state after pre-offsetting/kN	416291.9/416291.9
Components of the cable tension along the sliding surface on the side span side of the splay saddle in the unloaded state after pre-offsetting/kN	347276.3/393602.8
Components of the cable tension along the sliding surface on the anchor span side of the splay saddle in the unloaded state after pre-offsetting/kN	347276.3/393602.8

**Table 2:** Results of cable saddle pre-offsets adopting the method of Ref.^16^.

Offsets	Calculation results (left/right)
Offset of the tower saddles/m	2.3696/2.4248
Offset of the splay saddles/m	1.2578/1.8126
Horizon tension of the main cable on the main span side of the tower saddle in the unloaded state after pre-offsetting/kN	416289.5/416289.5
Horizon tension of the main cable on the side span side of the tower saddle in the unloaded state after pre-offsetting/kN	416289.5/416289.5
Components of the cable tension along the sliding surface on the side span side of the splay saddle in the unloaded state after pre-offsetting/kN	347269.3/347269.3
Components of the cable tension along the sliding surface on the anchor span side of the splay saddle in the unloaded state after pre-offsetting/kN	347269.3/347269.3

Comparing Tables 1 and 2, it can be seen that the pre-offsets of the tower and splay saddles calculated by the method of this paper are similar as those calculated by the method of Ref.^16^, but the method of Ref.^16^ needs to continuously try to calculate the balance conditions of the tower and splay saddles in the calculation process, and the calculation is cumbersome. Concurrently, it can also be seen that the pre-offsets of the cable saddles obtained by the above two methods can meet the balance conditions of the main and splay saddles.

## Conclusions

(1) An improved algorithm has been developed for the calculation of cable saddle pre-offsets, considering the coupling effect of tower and splay saddles. The algorithm deduced the nineteen-element nonlinear equations with the consideration of the mechanical equilibrium relationship and geometrical deformation conditions of the saddle and the main cable. Then, the tower and splay saddles pre-offsets were obtained by solving the above equations with the Newton–Raphson method, and the mechanics concept was clear and the solution simple.

(2) An initial value selection method was proposed for solving the nonlinear equations with the Newton–Raphson method. According to the position of the cable saddles in the completed state of the bridge, the slope of each point and the horizontal component force were obtained, which were used as the initial values for the calculation of the slope and the horizontal component force. The initial value of the tower saddle and splay saddle pre-offsets along the sliding surface could be taken as 1 m and 0.1 m, respectively.

(3) The analysed example showed that the pre-offsets of the cable saddles calculated by the improved algorithm could meet the balance conditions with high accuracy. The improved algorithm was suitable for the calculation of the pre-offsets of the saddles of the plane cable suspension bridge.

## Data Availability

All data, models, and code generated or used during the study appear in the submitted article.
